# Inflammation, dysregulated iron metabolism, and cardiovascular disease

**DOI:** 10.3389/fragi.2023.1124178

**Published:** 2023-02-03

**Authors:** Shaina L. Rosenblum

**Affiliations:** Department of Biochemistry, Jacobs School of Medicine and Biomedical Sciences, The University at Buffalo, Buffalo, NY, United States

**Keywords:** chronic inflammation, iron metabolism, heart iron, cardiovascular disease, cardiomyopathy

## Abstract

Iron is an essential trace element associated with both pathologic deficiency and toxic overload. Thus, systemic and cell iron metabolism are highly controlled processes regulated by protein expression and localization, as well as turnover, through the action of cytokines and iron status. Iron metabolism in the heart is challenging because both iron overload and deficiency are associated with cardiac disease. Also associated with cardiovascular disease is inflammation, as many cardiac diseases are caused by or include an inflammatory component. In addition, iron metabolism and inflammation are closely linked. Hepcidin, the master regulator of systemic iron metabolism, is induced by the cytokine IL-6 and as such is among the acute phase proteins secreted by the liver as part of the inflammatory response. In an inflammatory state, systemic iron homeostasis is dysregulated, commonly resulting in hypoferremia, or low serum iron. Less well characterized is cardiac iron metabolism in general, and even less is known about how inflammation impacts heart iron handling. This review highlights what is known with respect to iron metabolism in the heart. Expression of iron metabolism-related proteins and processes of iron uptake and efflux in these cell types are outlined. Evidence for the strong co-morbid relationship between inflammation and cardiac disease is also reviewed. Known connections between inflammatory processes and iron metabolism in the heart are discussed with the goal of linking inflammation and iron metabolism in this tissue, a connection that has been relatively under-appreciated as a component of heart function in an inflammatory state. Therapeutic options connecting inflammation and iron balance are emphasized, with the main goal of this review being to bring attention to alterations in iron balance as a component of inflammatory diseases of the cardiovascular system.

## Introduction

Cardiac disease is extremely complex. Both processes of inflammation and iron metabolism have been implicated in pathology of diseases of the cardiovascular system. Inflammation in the heart is well characterized, with upregulation of cytokines and inflammatory processes contributing to pathogenic changes. In cardiac disease, iron metabolism is less straightforward, with certain diseases involving heart iron accumulation and others iron deficiency. Many of the known pathways for iron handling in other cells have not been elucidated in cardiac cells, specifically cardiac endothelial cells. These cells are mostly ignored in heart iron metabolism research, which is surprising as they come in direct contact with systemic iron. Interestingly, iron metabolism and inflammation are deeply connected, but these connections are often not highlighted as a mechanism for disease pathogenesis. This review will discuss what is known about inflammation and iron metabolism in the heart and in heart disease, as well as highlight important areas of research that are missing in the current scope of the literature. Specifically, baseline processes of iron metabolism and inflammation in the heart are outlined, along with how these processes are altered in disease states. Connections between inflammation and iron metabolism in disease states are emphasized. Lastly, therapeutic options targeting both inflammation and iron metabolism are underlined, emphasizing alterations in iron handling as a mechanism for pathogenesis of inflammatory cardiac disease.

### Cellular and systemic iron metabolism

**FIGURE 1:**
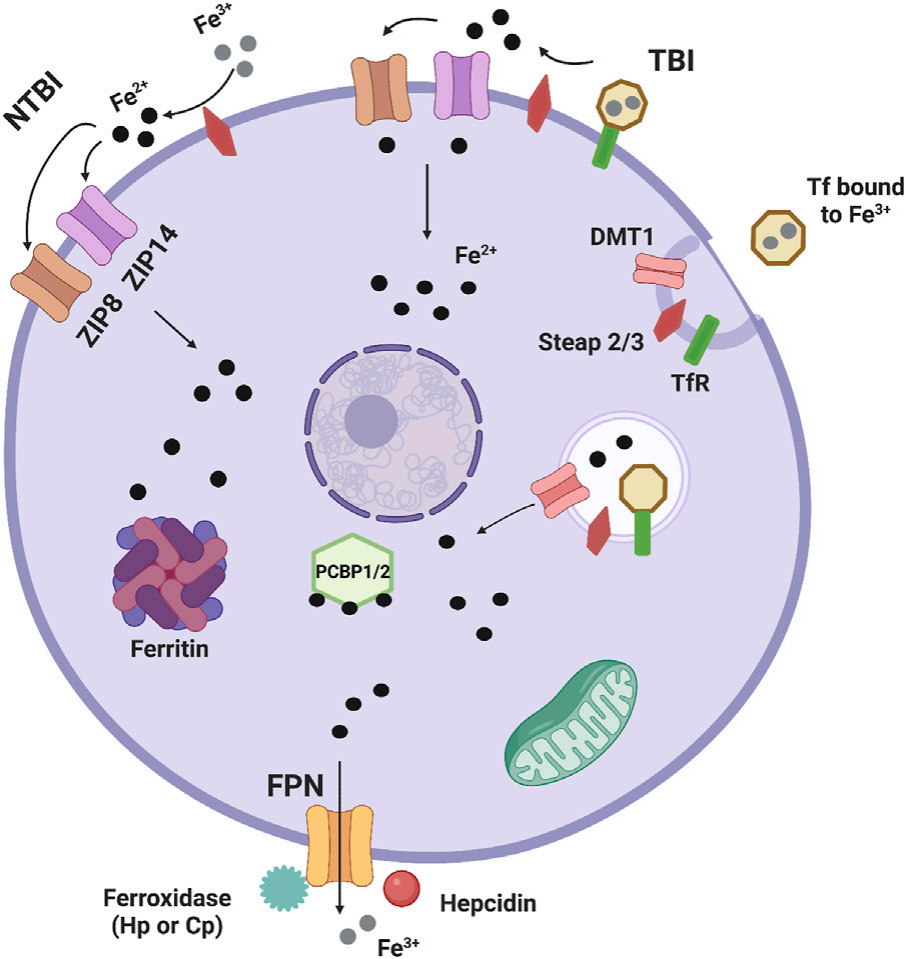
Basic cellular iron metabolism. This is an image showing basic mechanisms of iron handling in cells. Most iron is transported into tissue systems and cells through the canonical transferrin/transferrin receptor pathway. Transferrin (Tf) in serum binds to serum ferric iron (Fe3+), and then to a cell’s transferrin receptor (TfR). At this point, the complex is endocytosed. Then, iron is reduced by the ferrireductase Steap2/3, after which it can leave the endosome through the divalent metal transporter 1 (DMT1). Non-transferrin bound iron (NTBI) uptake also occurs. The first pathway involves reduction of extracellular ferric iron (Fe^3+^) by Steap2/3 and ferrous iron (Fe^2+^) entering the cell by either ZIP8 (*SLC39A8*) or ZIP14 (*SLC39A14*), two divalent metal ion transporters that can also transport Zn^2+^ and Mn^2+^. In another pathway, Fe^3+^ bound to Tf and the TfR is reduced by Steap2/3 and enters ZIP8 or ZIP14 for transport into the cell, highlighting that TBI can also be transported by ZIP8/14. Once the iron is within the cell, it can be shuttled to the iron storage protein ferritin by PCBP1/2 or taken to the mitochondria for iron-sulfur cluster and heme assembly. Iron efflux from cells is mediated by the efflux transporter ferroportin (FPN), which works with a ferroxidase, either ceruloplasmin (Cp) or hephaestin (Hp) to oxidize Fe^2+^ to Fe^3+^, allowing proper efflux. Lastly the acute phase protein hepcidin is well known to induce internalization of FPN leaving this protein non-functional and reducing iron efflux.

Iron is a transition metal essential to cell biology, and therefore whole-body wellness. Although essential, iron metabolism is a balancing act, as too much or too little can cause health issues throughout the body. As such, regulation of iron metabolism is tightly controlled. Cells have specific uptake and efflux mechanisms, along with intracellular iron handling chaperones. Iron transport from serum into tissue systems occurs at barriers, such as the blood-brain barrier, the blood-retinal barrier, the heart barrier, or the liver membrane, barriers that are tightly controlled. Ferric transferrin and the transferrin receptor are responsible for iron uptake by most tissue systems and cells. In the canonical pathway, Transferrin (Tf) in serum binds to serum ferric iron (Fe3+), and then to a cell’s transferrin receptor (TfR). At this point, the complex is endocytosed. Then, for iron to leave it must be reduced by the ferrireductase Steap2/3, after which it can leave the endosome through the divalent metal transporter 1 (DMT1) (Rouault, 2013). Alternatively, the Steap2/3-dependent reductive iron release from the Tf-TfR complex can occur at the plasma membrane with the Fe2+ transported into the cell *via* ZIP8 (*SLC39A8*) or ZIP14 (*SLC39A14*). These two divalent metal ion transporters are responsible also for non-transferrin bound iron (NTBI) uptake. This pathway involves reduction of extracellular ferric iron (Fe3+) by Steap2/3 followed by ZIP8/14 transport of Fe2+ (Kosman, 2020). Once the iron is within the cell, it can be shuttled to the iron storage protein ferritin by PCBP1/2 or taken to the mitochondria for iron-sulfur cluster and heme assembly (Shi et al., 2008). Iron efflux from cells is mediated by the efflux transporter ferroportin (FPN), which works with a ferroxidase, either ceruloplasmin (Cp) or hephaestin (Hp) to oxidize Fe2+ to Fe3+, allowing proper efflux (Figure 1).

Systemic iron is distributed throughout the circulation and within different organs. Most body iron is in the form of hemoglobin within circulating erythrocytes, about 2–3 g of total iron. Non-hemoglobin bound iron in the blood is around 2–3 mg of iron, bound to Tf. As mentioned previously, this TBI is sent to tissues for cellular iron use. Most iron in tissues is stored in ferritin within hepatocytes, macrophages in the liver, and red pulp macrophages in the spleen where it can be mobilized to blood plasma or other parts of the body during high iron demand (Ganz and Nemeth, 2012). Clearly the liver is the body’s largest iron store, but smaller amounts of iron are found in many other organs. As iron is necessary for Fe,S cluster formation and energy dynamics, organs that have high energy demands store more iron, such as the heart (Shuler et al., 1990) and brain (Beard and Connor, 2003; Nnah and Wessling-Resnick, 2018) (Wang et al., 2010). As this review focuses on the cardiovascular system, this will be elaborated on in coming sections.

Iron distribution in the body is highly regulated and can become dysregulated in the context of certain changes in homeostasis, such as inflammation. When an inflammatory stimulus occurs, monocytes and macrophages release cytokines, which trigger the release of acute phase proteins (APPs) from the liver. These proteins distribute throughout the body and cause downstream effects. Most notable to iron metabolism is hypoferremia, or loss of serum iron, suggesting that greater iron is retained in tissues (Gabay and Kushner, 1999). Several APPs are directly related to iron handling, connecting processes of inflammation and iron metabolism. During this response, serum Tf, an APP, decreases, suggesting less TBI uptake and possibly greater functioning of the non-canonical NTBI pathways. Other APPs of note that increase during this response include Cp and ferritin, likely relating to the increase in cellular iron (Gabay and Kushner, 1999). Most importantly, another APP, hepcidin, increases. Hepcidin is a hormone widely recognized as the master regulator of iron metabolism. This hormone is directly induced by the acute phase response, and more specifically the cytokine IL-6 (Nemeth et al., 2004a). Hepcidin regulates systemic iron levels by its direct actions on cellular FPN. Hepcidin binds to FPN and induces its internalization, blocking iron efflux (Nemeth et al., 2004b). This contributes to the idea, again, that during inflammation there is dysregulated iron homeostasis throughout the body. This review will focus on iron handling in the cardiovascular system, an understudied issue, with the goal of highlighting changes in iron metabolism as a possible mechanism for inflammatory cardiac disease.

## Iron metabolism in the heart

### Iron import mechanisms

Iron is important for heart function due to the high energy demands of constant muscle contraction. To understand iron accumulation in this tissue, the cell architecture of the heart must be described. Heart ion transport is mediated at the level of the vasculature, with endothelial cells (EC) comprising the ventricular and arterial blood vessel barriers. Heart vascular EC separate cardiomyocytes and other heart cell types from the systemic circulation, thus are the first heart cell to encounter circulating iron and inflammatory factors. Besides controlling solute transfer and providing protection, they control vasomotor tone and can regulate angiogenesis, two important properties for heart function (Aird, 2007). Iron accumulation often occurs at the level of the EC, as in the brain and the lungs (McCarthy and Kosman, 2015; Neves et al., 2019). The lung, brain, and heart all have a continuous, non-fenestrated endothelium structure, suggesting that iron accumulation may be similar between these tissues (Aird, 2007). Iron accumulation in the heart, like other ions, likely occurs in EC, which are the most abundant non-cardiomyocyte cell type in the heart (Pinto et al., 2016), although this accumulation has not been quantified. Mechanisms of iron accumulation into heart tissue are extremely important to understand, as both iron deficiency and iron overload can cause cardiovascular disease. Table 1 highlights iron metabolism proteins expressed in the cardiovascular system.

**TABLE 1:** Iron metabolism proteins expressed in cardiovascular system.

Protein	Protein function	Note	References
Transferrin/Transferrin Receptor 1 (Tf/TfR1)	Iron Uptake	Greater expression with iron loading, lower with chelation	Xu et al. (2015) Kasztura et al. (2017)
Divalent Metal Transporter 1 (DMT1)		Loss of DMT1 in mice reduces iron deposits and ROS	Ke et al. (2003), Davis and Bartfay (2004), Kumfu et al. (2011)
L-Type Calcium Channel (LTCC)		Blocking LTCC decreases cardiac iron levels	Tsushima et al. (1999), Oudit et al. (2006), Kumfu et al. (2016)
T-Type Calcium Channel (TTCC)		Present in development and resurfaces in cardiovascular disease	Kumfu et al. (2011) Huang et al. (2000) Kumfu et al. (2012)
ZRT/IRT-Like Protein 8/14 (ZIP8/14)		ZIP14 expressed highly in right and left ventricle and right atrium, ZIP8 expressed but at lower level	Kumfu et al. (2011) Begum et al. (2002); Taylor et al. (2005); Wang et al. (2012); Olgar et al. (2018a); Olgar et al. (2018b)
Ferroportin (FPN)	Iron Efflux	Greater expression with high iron, lower with chelation	Qian et al. (2007), Lakhal-Littleton et al. (2015), Berezovsky et al. (2022)
Hepcidin	Cellular and systemic iron regulation	Expression induced in hypoxic and inflammatory conditions	Merle et al. (2007), Isoda et al. (2010), Lakhal-Littleton et al. (2016)

**FIGURE 2:**
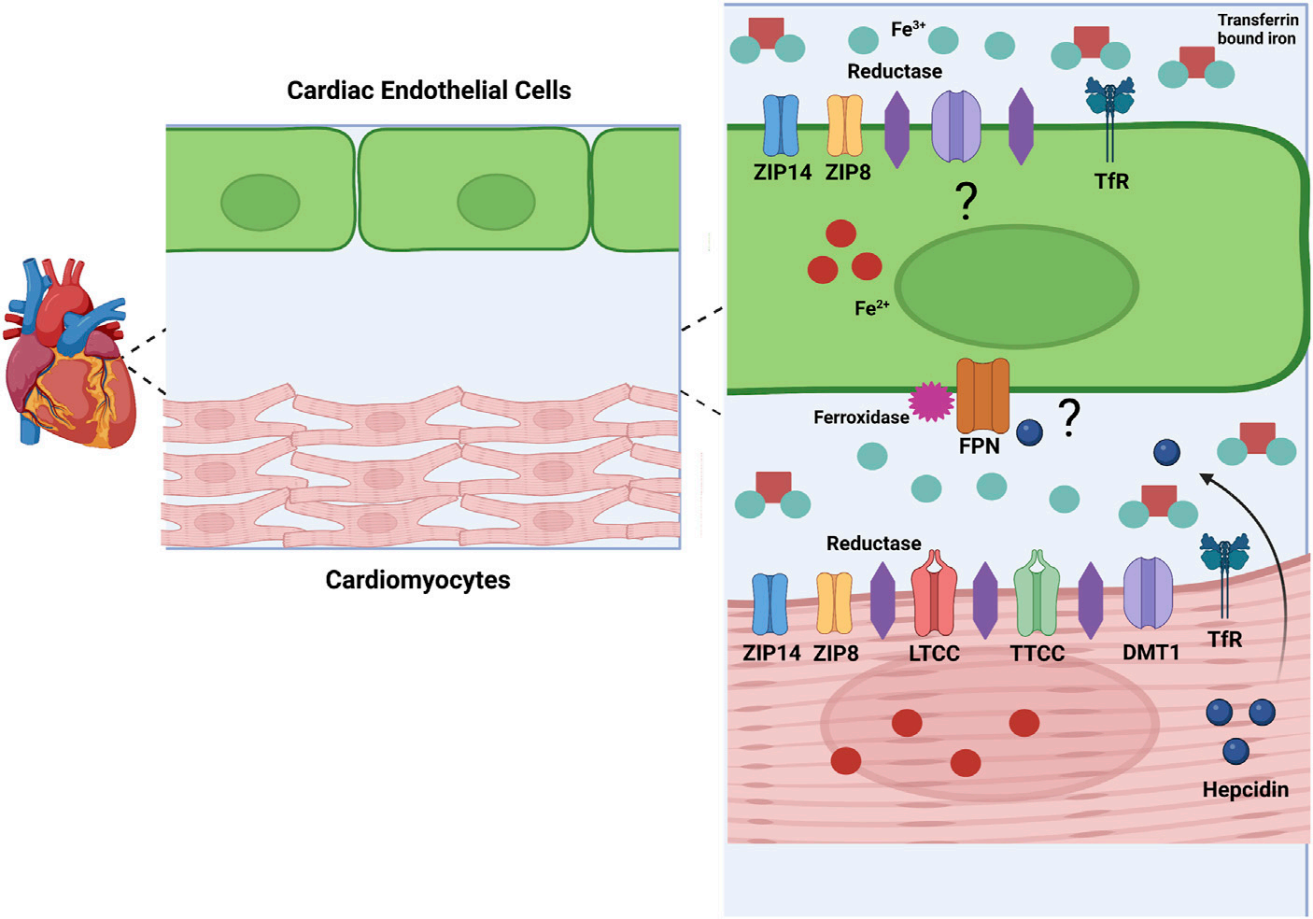
Model for Cardiac Iron Metabolism. This image represents a model of iron metabolism in cardiac tissue. Iron (Fe^2+^) enters the heart through cardiac vascular EC. Mechanisms of iron uptake in cardiac EC are unknown, but I propose involvement of ZIP8, ZIP14, DMT1, and the Tf/TFR pathway. Specifically, I propose that ZIP8 and ZIP14 can transport NTBI and TBI. Iron (Fe^2+^) leaves the EC through FPN and is oxidized by a ferroxidase to Fe^3+^. In the interstitial space, Fe^2+^ is reduced by a cardiomyocyte reductase and enters the cardiomyocyte through several possible transporters. ZIP14, ZIP8, LTCC, TTCC, DMT1, and the TfR pathway are all possible methods of iron uptake in cardiomyocytes. In conditions such as inflammation and hypoxia, hepcidin is produced in cardiomyocytes. As cardiomyocytes and EC are in paracrine contact, hepcidin could be released and alter FPN on the EC membrane, decreasing iron release from these cells. These are postulations, but more research needs to be done to confirm these methods of iron handling in cardiac cells. Created with Biorender.com.

As the heart’s main function is to circulate blood, cardiomyocytes are an important cell type in this tissue. Cardiomyocytes constitute about 75%–80% of cellular volume in the heart but come second to EC in cell number (Bergmann et al., 2015). Cardiomyocytes are known to import iron through many avenues (Figure 2). Cardiomyocytes import TBI through the canonical Tf/TfR pathway. The importance of TfR1 as a cardiomyocyte iron uptake transporter has been shown by the deletion of cardiomyocyte TfR1 in a mouse model. Mice lacking cardiomyocyte TfR1 experience lethal cardiomyopathy by the second week of age, due to abnormal mitochondrial morphology and function (Xu et al., 2015). TfR1 is expressed in the H9c2 rat cardiomyocyte line, and expression decreases with iron loading and increases with iron chelation, signifying the receptor’s role in iron handling in cardiomyocytes (Kasztura et al., 2017). More clinically relevant, patients undergoing treatment for advanced heart failure due to cardiomyopathy had significantly decreased levels of TfR1 and iron concentration in the left ventricle (LV), suggesting that this protein is important for healthy iron loading (Haddad et al., 2017). As part of the Tf/TfR canonical pathway, DMT1 is involved with iron uptake in the heart. Cardiac DMT1 protein levels in rats increased in iron deficiency and decreased in iron overload (Ke et al., 2003), while blocking DMT1 in mice reduces heart iron deposits, free radical production, and iron concentration, suggesting that DMT1 can help facilitate TBI iron uptake in this tissue (Davis and Bartfay, 2004; Kumfu et al., 2011).

There are several suggested methods of heart NTBI uptake (Figure 2). In 1999, Tsushima et al. found that L-type calcium channels (LTCC) involved in electrical excitability and ensuing calcium influx in cardiomyocytes may also be involved with NTBI uptake into rat hearts (Tsushima et al., 1999). In conditions of iron overload, calcium channels may be a method of NTBI accumulation resulting in cardiac dysfunction and iron overload cardiomyopathy. Blocking these channels can decrease cardiac iron levels and improve overall cardiac function (Oudit et al., 2006; Kumfu et al., 2016). Along with LTCC, T-type calcium channels (TTCC) are also suggested to be involved with NTBI in the heart. These channels are mostly present in heart development, but in conditions such as myocardial infarction (MI) or iron overload they resurface (Huang et al., 2000; Kumfu et al., 2012). Also, as with LTCC, blocking TTCC attenuates cardiomyocyte iron overload (Kumfu et al., 2011). Although these studies implicate LTCC and TTCC, there is little direct functional evidence that cardiomyocytes accumulate iron through these channels. The author’s work in the Kosman lab suggests that calcium increases localization of iron transporters to the brain EC plasma membrane. Therefore, this increased calcium influx from LTCC and TTCC could be contributing to iron uptake indirectly, through increased iron transporter membrane localization (Steimle et al., 2022). Another possible method for NTBI uptake into cardiomyocytes are the ZIP proteins, ZIP8 and ZIP14. ZIP14 is highly expressed in human heart tissue specifically in the left and right ventricle as well as the right atrium (Taylor et al., 2005). ZIP8 is also expressed in human heart tissue, but at a lower level than ZIP14 (Begum et al., 2002; Wang et al., 2012). Although NTBI uptake by ZIP transporters in the heart is not completely clear, some evidence suggests this occurs. In a study examining ß-thalassemic mice, iron accumulation and ZIP8 expression was increased in ventricular cardiomyocytes, suggesting involvement of this protein for NTBI and non-canonical TfR-dependent uptake from TBI in these cells (Kumfu et al., 2011). Increased expression of ZIP14 and decreased expression of ZIP8 was found in heart tissue from human patients with heart failure and heart failure-induced rat cardiomyocytes. This study focused on the uptake of zinc, showing that this was greater in heart failure, correlating to the increase in zinc transporters, but NTBI uptake in this case was not examined (Olgar et al., 2018a). Another study from the same lab used a rat hypertrophic heart model and found similarly increased levels of ZIP14 and decreased ZIP8, along with an increased zinc concentration in hypertrophic cardiomyocytes (Olgar et al., 2018b). It is well known that the ZIP proteins can transport iron as well as zinc, therefore it is likely that zinc transporters play a role in NTBI in cardiomyocytes, although this idea has never been functionally tested. Overall, mechanisms of cardiomyocyte NTBI need to be thoroughly examined.

### Iron export and regulation

The only known iron export protein, FPN, is also found in the heart. In adult mouse heart FPN was found in cardiomyocytes within the ventricular myocardium. FPN was localized to the cell membrane and was also present in the intracellular regions of some cardiomyocytes. In conditions of high iron, FPN expression increased; iron chelation resulted in a knock-down of FPN (Lakhal-Littleton et al., 2015). Other studies complimented this result by showing that FPN expression changes similarly in response to low and high iron diets in rats (Qian et al., 2007; Berezovsky et al., 2022). Cardiomyocyte-specific deletion of FPN in mice results in a low survival rate, with a median lifespan of 22 weeks. Mouse hearts were examined and left ventricular enlargement, thinning of the left ventricular wall, and altered cardiomyocyte morphology was found. Also, impaired cardiac performance coincided with increased total iron concentration (Lakhal-Littleton et al., 2015). These results identify FPN as a key protein for proper cardiac iron metabolism and critical for cardiac functioning.

As noted above, hepcidin is the master regulator of systemic iron metabolism. Hepcidin is expressed in the rat myocardium within the intercalated disc region and expression is induced by hypoxic and inflammatory conditions (Merle et al., 2007). HAMP, the human gene name for hepcidin, is expressed in the adult mouse heart, at a 30-fold lower rate than in the liver. With regards to iron regulation of HAMP, mRNA levels were decreased with a low iron diet and increased with an iron overloaded diet (Lakhal-Littleton et al., 2016). Hepcidin is also expressed in the human heart with expression elevated in myocarditis and myocardial infarction (Isoda et al., 2010). Data indicate that hepcidin plays a role in local heart iron handling and cardiac function. Evidence for this premise comes from a study using a mouse model with a cardiomyocyte specific deletion of the HAMP gene and a cardiomyocyte specific knock-in of FPN housing a mutation that prevents appropriate hepcidin binding (Lakhal-Littleton et al., 2016). Similar to the work on cardiomyocyte deletion of FPN, cardiomyocyte deletion of HAMP resulted in a low survival rate (median of 28 weeks), enlarged left ventricles, hypertrophic cardiomyocyte morphology, and a reduction in left ventricle ejection fraction (LVEF), a measure of proper cardiac function. The mice carrying the mutation that prevents hepcidin binding exhibited similar pathophysiology. Importantly, in both mouse models, FPN expression was upregulated, and iron efflux increased, suggesting that increased FPN *function* led to cardiac dysfunction with cardiac loss of hepcidin (Lakhal-Littleton et al., 2016). These results emphasize the importance of the hepcidin/FPN axis for favorable cardiovascular function.

Although no direct evidence exists supporting expression of FPN or hepcidin in EC, these proteins likely are involved with iron export and regulation in this barrier system. Although research has focused exclusively on cardiomyocytes, EC are the first cells to encounter systemic iron and inflammatory signals and therefore would require proper efflux machinery, including FPN, to deliver systemic iron to the myocardium. The fact that cardiomyocytes express hepcidin suggests that EC FPN function would be under control of this paracrine regulator. Also, EC may express hepcidin themselves thus regulating heart iron accumulation in an autocrine fashion, as in the corneal endothelium of the eye (Ashok et al., 2020) a possibility that requires experimental examination.

## Inflammation and inflammatory diseases of the cardiovascular system

### Inflammation in the cardiovascular system

Immune cells in the heart either reside in the tissue permanently, such as macrophages and dendritic cells, or infiltrate when necessary, such as B cells and T cells. Other immune cells in heart tissue include monocytes, eosinophils, mast cells, and neutrophils (Swirski and Nahrendorf, 2018; Strassheim et al., 2019). Crosstalk between heart immune cells and cardiomyocytes, EC, and fibroblasts occurs, regulating how the immune cells respond and contribute to homeostasis. The cardiac immune system is as important to maintain homeostasis as it is during disease and injury. For example, yolk-sac derived macrophages are essential for maturation of the cardiovascular system, as they remodel the coronary plexus and expand the vasculature during this stage of development (Leid et al., 2016). At homeostasis macrophages are found in the interstitial space of the myocardium and closely associate with EC and cardiomyocytes, highlighting participation in endocrine and paracrine signaling necessary for maintaining homeostasis (Pinto et al., 2012). During steady state physiology, macrophages are found in the left ventricular myocardium in the human heart. In mouse hearts, it has been shown that during steady state, turnover of macrophages in the myocardium is very slow, but that these macrophages perform their normal functions of phagocytosing pathogens. Also, evidence suggests that local proliferation of macrophages is more prominent than monocyte infiltration from the blood during a normal physiological state (Heidt et al., 2014). Myocarditis, or heart inflammation, and how the myocardium responds to inflammation in a disease state is outlined in Table 2 and discussed in the following sections.

**TABLE 2:** Evidence of inflammation in cardiovascular disease.

Condition	Evidence of inflammation	Effect on heart function	References
Heart Failure	Elevated pro-inflammatory cytokine levels	LV dysfunction, pulmonary edema, LV remodeling, cardiomyopathy	Perticone et al. (2019) Levine et al. (1990) Torre-Amione et al. (1996); Vasan et al. (2003); Latini et al. (2012); Edelmann et al. (2015); Mann (2015) Pagani et al. (1992); Kubota et al. (1997); Bozkurt et al. (1998)
	Decreased expression of protective IL-10	Reduced NOS in EC, decreased blood flow	Perticone et al. (2019)
Cardiomyopathy	Elevated serum IL-6R, IL-8, MCP1 and MIP1β	Disruption of desmosomal junctional proteins	Asimaki et al. (2011)
	Increased myocardial IL-17 and TNF⍺		Asimaki et al. (2011)
	CD45^+^ and CD43^+^ lymphocyte infiltration	Ventricular arrythmia, ventricular enlargement, sudden death	Basso et al. (1996)
	Expression of autoantibodies against desmosomal cadherin proteins	Cardiomyocyte gap junction dysfunction	Chatterjee et al. (2018)
	Elevated TNF⍺, IL-6, IL-8, IL-1β, and CRP	Higher risk of MI, stroke, cardiac death, LV diastolic dysfunction	Soinio et al. (2006), Bruno et al. (2009), Dinh et al. (2009), Herder et al. (2011), Ramesh et al. (2022)
	RAAS induced NF-κB expression	Diastolic dysfunction, hypertension	Sciarretta et al. (2009)
	NLRP3 inflammasome activation and IL-1β release	LV dysfunction, myofibril destruction, metabolic deficits	Van Tassell et al. (2013)
Myocardial Infarction	Higher IL-2, IL-6, IL-8, IL-10, GM-CSF and TNF-⍺ levels	Increased risk of heart failure after MI, unstable angina	Satoh et al. (2006) Methe et al. (2005)
	NLRP3 inflammasome activation and resulting IL-1β, IL-18 expression	Greater infarct area, myocardial fibrosis, ventricular remodeling, cardiomyocyte death	Venkatachalam et al. (2009), Kawaguchi et al. (2011), Mezzaroma et al. (2011), Sandanger et al. (2013)
	NF-κB expression found near infarct zone	Increased chemokine expression, infiltrating inflammatory cells	Chandrasekar and Freeman (1997), Chandrasekar et al. (2001), Hilfiker-Kleiner et al. (2010)

### Inflammation and aging

With increasing age, the prevalence of cardiovascular disease increases, and is the leading cause of death in people aged greater than 65 years (Rosamond et al., 2008). In general, with aging, inflammation increases. Low grade inflammation characterized by increased levels of circulating pro-inflammatory cytokines contributes to many age-related diseases. Several theories of aging involve heightened inflammation, including the free radical theory, the cellular senescence theory, and the network theory of aging (Franceschi et al., 2000; MacNee et al., 2014). Chronic inflammatory diseases often appear with aging and age is a major risk factor for these diseases. Specifically, many cardiovascular diseases are prevalent with aging such as the three discussed below, heart failure, cardiomyopathy, and MI (Dhingra and Vasan, 2012; Livshits and Kalinkovich, 2019). Aging stimulates structural and functional changes in the heart. Structurally, these changes encompass increased arterial ventricular thickness, a loss of the endothelial barrier, fibrosis, and changes in the extracellular matrix (ECM). Functionally, this results in a lower heart rate, greater chance of arrythmia, and loss of cardiac relaxation, or diastolic function (Obas and Vasan, 2018). Molecular mechanisms related to these structural and functional changes may involve or be caused by age-related alterations in inflammatory processes. Also of interest, aging and the resulting inflammation can affect iron handling in the cardiovascular system. Anemia of inflammation can occur in aging, in which it is termed anemia of aging. In this disease, there are high levels of circulating cytokines, with the most important being IL-6. This results in greater expression of hepcidin and less serum iron, or hypoferremia (Nemeth and Ganz, 2014). Interestingly, iron accumulation also can occur with aging. Often, cardiovascular diseases associated with aging and inflammation involve iron overload that contributes to pathogenesis, as discussed in the following sections. In the following sections, inflammatory and iron handling processes will be discussed with respect to cardiac disease, and it is important to remember that these diseases are more prevalent with increasing age.

### Heart failure

Heart failure is a clinical syndrome characterized by cardiac structure abnormalities and functional impairment including left ventricle filling and/or ejection problems. Heart failure is one of the most common causes of recurring trips to the hospital and death (Kemp and Conte, 2012; Ponikowski et al., 2016; Perticone et al., 2019). A link between heart failure and inflammation was first described by Levine et al., when they discovered that patients with heart failure with reduced ejection fraction (HF-rEF) also had increased TNF levels (Levine et al., 1990). Since this finding, several studies have shown that heart failure is marked by elevated pro-inflammatory cytokines circulating in the serum. Elevated levels of TNF⍺, IL-6, CRP, Galectin-3, IL-2, IL-1β, IL-8, IL-33, and pentraxin-3 are all found in patients with heart failure (Torre-Amione et al., 1996; Vasan et al., 2003; Latini et al., 2012; Edelmann et al., 2015; Mann, 2015). Cytokine overexpression in heart failure correlates with a heart failure “phenotype”, including LV dysfunction, pulmonary edema, LV remodeling, and cardiomyopathy (Pagani et al., 1992; Kubota et al., 1997; Bozkurt et al., 1998). This has led some to believe in the cytokine hypothesis of heart failure, suggesting that increased cytokine expression may be a mechanism for this disease progression. Of interest, different classes of heart failure can emulate differing inflammatory profiles. Heart failure can present with reduced ejection fraction or preserved ejection fraction. With respect to inflammatory markers, HF-rEF had significantly higher circulating levels of all pro-inflammatory cytokines associated with heart failure and a reduction in the protective cytokine IL-10, suggesting a greater inflammatory milieu in this type of heart failure (Perticone et al., 2019). The CANTOS (Canakinumab Anti-Inflammatory Thrombosis Outcome Study) trial began to study the inflammation hypothesis of thrombosis and atherosclerosis. This study examined the effects of a monoclonal antibody against IL-1β, Canakinumab, on atherosclerotic events in patients with high levels of CRP and previous history of MI. This trial discovered that patients given the 150 mg dose of Canakinumab every 3 months for 5 years had 15% lower risk of primary cardiovascular events than placebo (Ridker et al., 2017). Patients from this trial were also utilized to study the effects of Canakinumab on hospitalizations for heart failure, as inflammation is associated with increased risk of heart failure and adverse prognosis following heart failure. This study found that increasing doses of Canakinumab resulted in less hospitalizations for heart failure and heart failure-related mortalities in patients with prior MI and high CRP levels (Everett et al., 2019). These studies and results highlight that inflammatory cytokines and processes are involved with diseases of the cardiovascular system and can be targeted to prevent these diseases. Along with effects on the myocardium, inflammatory mediator profiles in heart failure can also affect EC. Serum from patients with severe heart failure can reduce expression of endothelial nitric oxide synthase (NOS), resulting in increased apoptosis in human umbilical vein EC, due to levels of TNF⍺ (Agnoletti et al., 1999). Also, higher circulating levels of TNF⍺ can decrease blood flow in heart failure patients (Anker et al., 1998), further noting the changes that cytokines can have on heart EC and overall heart function with heart failure. Endothelial interaction with inflammatory mediators highlights why EC should be further studied with respect to iron metabolism.

### Cardiomyopathy

The American Heart Association (AHA) recommends the use of a classification system that defines cardiomyopathy as primary or secondary. In primary cardiomyopathy, disease progression is solely due to changes in the heart, while secondary cardiomyopathy occurs from a separate systemic condition (Maron et al., 2006). One example of a primary form is arrhythmogenic cardiomyopathy (ACM), predominantly caused by genetic defects in desmosomes connecting cardiomyocytes in the intercalated discs. This disease is characterized by electrical instability, life threatening arrhythmias, and increased risk of sudden cardiac death (Corrado et al., 2020). Also, in patients with ACM, elevated serum levels of IL-6R, IL-8, MCP1 and MIP1β are found along with increased myocardial expression of IL-17 and TNF⍺ (Asimaki et al., 2011). In one study, areas of inflammatory infiltrates were found in ¾ of postmortem human hearts with ACM. These patchy inflammatory infiltrates consisted of CD45^+^ and CD43^+^ lymphocytes, which were associated with cardiomyocyte death in 67% of ACM hearts. Electron microscopy showed that these infiltrates were found near capillary vessels, as well as evidence of cardiomyocyte debris within the fibrotic regions (Basso et al., 1996). Another aspect of inflammation found in several cardiomyopathies is autoimmunity and the presence of autoantibodies. Autoantibodies against desmosomal cadherin proteins were discovered in patients with ACM, likely explaining cardiac inflammation that occurs in this disorder (Chatterjee et al., 2018).

An example of a secondary cardiomyopathy that is associated with inflammation is diabetic cardiomyopathy (DCM). This disease was first described in 1972 by Rubler et al., who found enlarged hearts with congestive heart failure, LV hypertrophy and myocardial fibrosis in 4 diabetic patients (Rubler et al., 1972). In 1974, the Framingham Heart Study showed that the risk of heart failure was five times higher in people with type 2 diabetes (T2D) (Kannel et al., 1974). T2D and DCM include markers of inflammation such as TNF⍺, IL-6, IL-8, IL-1β, and C-reactive protein (CRP) (Soinio et al., 2006; Bruno et al., 2009; Dinh et al., 2009; Herder et al., 2011; Ramesh et al., 2022). Another pathway activated in diastolic dysfunction associated with DCM is the renin-angiotensin-aldosterone system (RAAS). In T2D, RAAS activity is amplified and evidence suggests that angiotensin-2, a mediator in the RAAS pathway, can activate NF-κB resulting in myocardial inflammation (Sciarretta et al., 2009). The NLRP3 inflammasome has been implicated in inflammation seen in DCM. NLRP3 inflammasomes are activated in hearts of rats with T2D, through NF-κB activation (Luo et al., 2014). Activation of the NLRP3 inflammasome results in IL-1β release through a caspase-1 mechanism, which causes impaired cardiac contractility in mice (Van Tassell et al., 2013). The commonality between all these inflammatory mediators linking diabetes to cardiomyopathy is NF-κB activation.

### Myocardial infarction

Myocardial infarction occurs when there is a decrease or stoppage of blood flow to a region of the heart, often due to a blockage, or ischemia. This results in necrosis of the heart muscle. Necrotic cells activate the innate immune system triggering a strong inflammatory response characterized by stimulation of toll-like receptors (TLRs) and complement protein release (Prabhu and Frangogiannis, 2016). During an ischemic event, hypoxia impairs EC integrity and barrier function, increasing leukocyte infiltration into the heart (Eltzschig and Eckle, 2011). Stressed or necrotic cells then release danger-associated molecular patterns (DAMPs) that bind to pattern recognition receptors (PRRs) and activate release of inflammatory cytokines, chemokines, and cell adhesion molecules (Arslan et al., 2011; de Haan et al., 2013). Aside from release of DAMPs from leukocytes, they can also be released from stressed cardiomyocytes and fibroblasts (Yamauchi-Takihara et al., 1995; Gwechenberger et al., 1999). Increased TLR4 activation and downstream pro-inflammatory mediator signaling has been seen in humans with acute MI. Higher levels of IL-2, IL-6, IL-8, IL-10, GM-CSF and TNF-alpha were found in acute MI patients compared to healthy subjects (Methe et al., 2005; Satoh et al., 2006). Components of the NLRP3 inflammasome and the end-products of inflammasome activation, IL-1β and IL-18 are upregulated after MI in several cell types including EC, fibroblasts, and cardiomyocytes near the MI border zone (Venkatachalam et al., 2009; Kawaguchi et al., 2011; Mezzaroma et al., 2011; Sandanger et al., 2013). Patients with acute MI display greater activation of NF-κB from circulating leukocytes (Ritchie, 1998; van der Pouw Kraan et al., 2014). In rodents undergoing myocardial ischemia, NF-κB activation is found near the infarct zone (Chandrasekar and Freeman, 1997; Chandrasekar et al., 2001). Several studies highlight the importance of cytokines to the inflammatory response after MI. First, mice with mutant gp130, the receptor for IL-6, have lower IL-6 levels, less sustained inflammation, and a lower mortality rate with MI (Hilfiker-Kleiner et al., 2010). Also, mice with TNF knockout or treated with an anti-TNF antibody who had undergone ischemia/reperfusion displayed a decreased infarct area in the LV, less arrhythmia, less NF-κB activation, and a reduction in cytokine expression (Maekawa et al., 2002). Overall, many lines of evidence suggest that a strong inflammatory response occurs in MI and that this response augments outcomes related to cardiovascular function.

## Inflammation and iron metabolism in the cardiovascular system

### Connections between systemic inflammation and iron metabolism

Inflammation is initiated by changes in the body’s homeostasis such as viral infection, bodily injury, or immunological disorders. When inflammation is triggered, monocytes are activated and release cytokines that induce the synthesis and release of APPs from hepatocytes. Once released, these factors can travel to various tissues and initiate downstream effects relating to an inflammatory response, such as fever and leukocytosis (Gabay and Kushner, 1999). Importantly, there are several APPs that are directly related to iron homeostasis. For example, concentrations of ferritin and ceruloplasmin increase during acute phase inflammation, while levels of transferrin decrease. An important downstream effect of inflammation is a decrease in the serum concentration of iron, or hypoferremia, signifying iron sequestration in cells and tissue systems (Weiss, 2009). This can result in anemia of inflammation, which encompasses many other defining features including suppression of erythropoiesis, erythrocyte destruction, and a switch to leukopoiesis in the bone marrow (Nemeth and Ganz, 2014). Hypoferremia is due to another critical APP, hepcidin. As mentioned previously, hepcidin binds directly to the iron efflux protein FPN, inducing its internalization, and targeting this protein for ubiquitination (Nemeth et al., 2004b). The cytokine IL-6 directly upregulates expression and release of hepcidin from certain body stores and cells, most notably the liver (Nemeth et al., 2004a). This results in iron overloaded cells and tissues, with less iron delivery to other parts of the body. Therefore, inflammatory responses have direct effects on systemic and tissue iron metabolism.

### Cellular impacts of iron dysregulation in cardiomyocytes

How cardiomyocytes are altered in cardiovascular disease associated with iron dysregulation is not fully understood, though some evidence hints at basic cell impacts. In general, this section will focus on effects of iron dysregulation on cardiomyocyte oxidative stress, mitochondrial integrity, energy metabolism, and ferroptosis. Several studies provide evidence that iron induces oxidative stress in cardiomyocytes. Treatment of H9c2 cardiomyocytes with iron induced generation of reactive oxygen species (ROS) that correlated to increased insulin resistance and improper clearance by autophagy (Sung et al., 2019). Another study elucidated that treatment of cardiomyocytes with iron resulted in increased mitochondrial ROS that correlated to mitochondrial membrane depolarization, suggesting mitochondrial dysfunction with iron overload (Gordan et al., 2020). Interestingly, indirectly changing cell iron status by downregulating ferritin heavy chain (FHC) also alters cardiomyocyte oxidative stress. Cardiomyocytes infected with adenovirus knocking down FHC showed a greater labile iron pool, greater levels of ROS markers, and lower cell survival (Omiya et al., 2009). Overall, these studies associate changes in oxidative stress with iron dysregulation in cardiomyocytes.

Ferroptosis is an iron-dependent form of cell death characterized by redox stress due to iron-induced lipid peroxidation, depletion of glutathione, and inactivation of glutathione peroxidase 4 (GPX4) (Yang and Stockwell, 2016). This form of cell death occurs in many cardiovascular diseases, as will be discussed in the coming sections, but the cellular mechanisms in cardiomyocytes, or other cardiovascular cell types are not well characterized. In 2018, Baba et al. (2018), showed that inducing ferroptosis in cardiomyocytes significantly increased cell death and ROS production, which was alleviated by treatment with Ferrostatin-1, an ROS scavenger. Machado et al. (2022), in 2022 examined if FHC deletion in cardiomyocytes altered mitochondrial dynamics and cardiac function. Interestingly, they found that deletion of FHC did not affect mitochondrial or overall cardiac function, but rather the expression of genes related to glutathione metabolism, including SLC7a11 and heme oxygenase 1 (HO-1). Using mice that overexpress HO-1, they elucidated that greater HO-1 expression increases expression of SLC7a11, a cysteine exchanger, which results in greater intracellular cysteine, glutathione, and overall lower risk of ferroptosis. These results suggest that without FHC there is a compensatory increase in transcriptional processes leading to an antioxidant effect in cardiomyocytes, linking iron dysregulation to cellular changes in cardiomyocytes.

Iron dysregulation in cardiomyocytes can alter mitochondrial integrity and energy metabolism. Mitochondrial membrane potential is a measure of mitochondrial health because it represents the process of electron transport and oxidative phosphorylation. When membrane potential decreases, this indicates loss of mitochondrial integrity. Several studies have shown decreased mitochondrial membrane potential in the presence of iron (Chan et al., 2015; Akentieva et al., 2019; Gordan et al., 2020). Fang et al. found that during cardiomyocyte ferroptosis, lipid peroxidation occurs most in mitochondrial membranes, altering mitochondrial integrity and affecting mitochondrial dynamics (Fang et al., 2019). Interestingly, in another study, iron deficient cardiomyocytes appeared swollen and contained electron dense bodies harboring sulfur-based proteins. These structural deficits were correlated to changes in energy metabolism including a loss of ATP and ATP-linked respiration and loss of electron transport complex enzymatic activity (Hoes et al., 2018). In the case of Friedrich’s Ataxia (FRDA), a disease characterized by cardiomyopathy, mitochondrial and energy metabolism alterations are disease mechanisms. Induced pluripotent stem cells (iPSCs) from a human patient with FRDA were obtained and differentiated into cardiomyocytes. These cells displayed greater iron accumulation, and energy metabolism deficits seen by reduced ATP synthesis in the presence of iron, suggesting that iron affects cardiomyocyte energy synthesis. Altogether, both overloaded and iron deficient cardiomyocytes have mitochondrial abnormalities that result in dysregulated energy metabolism.

### Connections between inflammation and iron metabolism in cardiovascular disease

Cardiovascular diseases are clearly altered by a strong inflammatory response. Several lines of evidence suggest that iron handling in these diseases is abnormal as well (Table 3). In the case of heart failure, anemia and iron deficiency alter disease progression. Data from the OPTIME-CHF study in 2003 revealed that anemia is common in patients hospitalized with decompensated heart failure and that decreasing hemoglobin levels were associated with greater risk (Felker et al., 2003). Anemia was shown to be a risk factor for development and rehospitalization of heart failure in patients with end-stage renal disease (ESRD) (Al-Ahmad et al., 2001). Nanas et al. (2006), investigated a group of patients with end stage heart failure and found that iron deficiency anemia was the most common cause of anemia at 73%, which correlated to low iron stores measured in bone marrow biopsies. Mechanisms for this iron deficiency anemia in heart failure are contested, but evidence reports relations to inflammatory cytokines. The BIOSTAT-CHF study measuring risk factors in patients with heart failure (Voors et al., 2017), found elevated IL-6 levels in 56% of patients, significantly correlating to anemia, reduced LVEF, and poorer clinical outcomes (Markousis-Mavrogenis et al., 2019). Also using data from the BIOSTAT-CHF cohort, van der Wal et al. (2019), correlated high CRP levels to iron deficiency. Conflicting results have been found with respect to hepcidin levels in heart failure (Anand and Gupta, 2018). Perticone et al. (2019), found differing levels of hepcidin in different types of heart failure. In heart HF-rEF hepcidin levels were significantly increased, while in heart failure with preserved ejection fraction (HF-pEF), hepcidin levels decreased. In the BIOSTAT-CHF cohort, patients with iron deficiency anemia had the lowest levels of hepcidin, despite measuring increased expression of inflammatory cytokines. This study suggests that these patients have lower hepcidin levels possibly due to changes in systemic iron availability rather than induction of inflammation (van der Wal et al., 2019). This idea was first described by another study in which heart failure patients with iron deficiency anemia had lower hepcidin levels, despite high TNF⍺ levels, suggesting that iron status affects hepcidin more than inflammation does (Weber et al., 2013). Based on these results, more research needs to be completed in this field to determine the mechanisms behind the differences in iron regulation in heart failure.

**TABLE 3:** Evidence for alterations in iron metabolism in cardiovascular disease.

Condition	Change in iron metabolism	References
Heart Failure	Decreased hemoglobin associated with increased risk for heart failure	Felker et al. (2003)
	Iron deficiency anemia associated with heart failure	Al-Ahmad et al. (2001) Nanas et al. (2006)
	Increased hepcidin in HF-rEF, decreased in HF-pEF	Perticone et al. (2019)
Myocardial Infarction	Increased iron at infarct region	Moon et al. (2020)
	Greater hepcidin levels in rat model of acute MI	Isoda et al. (2010)
	Ferroptosis-induced cell death occurs in MI	Fang et al. (2019) Park et al. (2019) Liu et al. (2021)
Cardiomyopathy	Iron deposition in ventricular and atrial myocardium	Wood (2008)
	Increased iron accumulation in cardiomyocytes leading to ROS	Kremastinos and Farmakis (2011)
	Ferroptosis-induced cell death as mechanism for cardiomyopathy	Wang et al. (2022) Goldin et al. (2006)

Myocardial infarction is characterized by several changes in iron-related processes. Quantitative susceptibility mapping (QSM)-MRI, which is used to measure tissue iron content, found increased iron at regions of myocardial infarct and greater tissue iron staining in a swine model of MI (Moon et al., 2020). In a rat model of acute MI, hepcidin and IL-6 transcripts were significantly upregulated, suggesting a connection between inflammation and iron regulation in this disease (Isoda et al., 2010). Many studies emphasize the role that ferroptosis plays in MI. Mice experiencing myocardial ischemia displayed higher levels of cardiac non-heme iron, greater infarct size, and increased levels of ferroptosis markers compared to sham mice, which was rescued when treating with the ferroptosis inhibitor ferrostatin-1 (Fer-1) (Fang et al., 2019). In a mouse model of MI, left anterior descending ligation (LAD), mRNA and protein levels of GPX4 were downregulated. In this same study, gene ontology showed that interferon gamma (IFN-Ɣ) and IL-2 signaling was enriched during the first week after MI (Park et al., 2019). Weighted gene coexpression network analysis (WGCNA) was used to determine key genes involved in acute MI, ferroptosis, and hypoxia. This analysis found that NF-κB, MyD88, and IL-1β were all upregulated, which correlated to an increase in these genes in acute MI mouse myocardial tissue (Liu et al., 2021). These results connect ferroptosis, an iron-related cell death, to inflammatory processes in MI and highlight ferroptosis as a potential therapeutic target in cardiovascular disease.

Cardiomyopathy is often associated with iron overload. Iron overload cardiomyopathy is an aspect of several diseases with cardiovascular deficits including T2D and thalassemia. Pathologically this disease is characterized by dilated cardiomyopathy with left ventricular systolic dysfunction (Horwitz and Rosenthal, 1999). Iron deposition occurs initially in the ventricular myocardium and later in the atrial myocardium (Wood, 2008). Iron accumulated in cardiomyocytes is either stored in ferritin or is free iron, leading to increased ROS due to the Fenton reaction (Kremastinos and Farmakis, 2011). Interestingly, increased iron accumulation through the LTCC in cardiomyocytes results in abnormal calcium transport and impaired excitation-contraction coupling, furthering cardiac dysfunction (Murphy and Oudit, 2010). Recently, lipocalin-2 (Lcn2) an inflammatory mediator, has emerged as playing a role in iron overload cardiomyopathy. Lcn2 is a molecule that sequesters iron from bacteria as part of the anti-bacterial iron depletion process of innate immunity (Goetz et al., 2002). Mice treated with Lcn2 have increased macrophage infiltration into cardiomyocytes, leading to apoptosis. *In vitro* results from the same study using H9c2 cells and primary neonatal rat cardiomyocytes found that Lcn2 induced iron accumulation, cleaved caspase-3 activity, and mitochondrial membrane depolarization, suggesting that Lcn2 induces apoptosis by an inflammation and iron-dependent process (Xu et al., 2012). Cell death induced by ferroptosis is associated with T2D and DCM. Research in engineered cardiac tissue from neonatal mouse ventricular heart cells illustrates that advanced glycation end products (AGEs) induce ferroptosis (Wang et al., 2022). AGEs are also known to induce NF-κB nuclear translocation and transcription of several inflammatory mediators including pro-inflammatory cytokines and cell adhesion molecules, ultimately creating an inflammatory response (Goldin et al., 2006). Therefore, it may be possible that AGEs activate ferroptosis through an inflammatory method, but more research is needed to understand this. Other research connecting ferroptosis and inflammation in DCM found that activation of the NRF2 pathway attenuated AGE-induced ferroptosis (Wang et al., 2022). Activating the NRF2 pathway is known to be anti-inflammatory through regulation of HO-1 and NF-κB signaling, suggesting an inverse relationship between NRF2 and inflammation (Saha et al., 2020). Based on this information, it may be that ferroptosis in DCM is regulated by inflammatory processes, but this has not been fully elucidated. Interestingly, chemotherapy cancer treatment has been shown to promote cardiomyopathy resulting in doxorubicin-induced cardiomyopathy (DIC), through ferroptotic cell death. Mice treated with doxorubicin displayed decreased GPx4, increased amounts of lipid peroxidation, and greater cell death by TUNEL staining. In the same study, cultured cardiomyocytes were treated with doxorubicin and were found to have less total and mitochondrial GPx4. Cell survival and lipid peroxidation were rescued using Fer-1, a ferroptosis inhibitor, suggesting that ferroptosis plays a role in the cardiotoxicity induced by chemotherapies (Tadokoro et al., 2020). As cancer can both be caused by inflammation and perpetuate inflammatory processes, this further connects inflammation and iron metabolism in cardiac disease.

## Treatment options: Iron metabolism and inflammatory cardiovascular disease

Cardiovascular disease involves processes linked to inflammation and iron metabolism, but research on how this link can be targeted therapeutically is limited. The discussion above supports the premise that iron-related processes can be a key component in inflammatory aspects of cardiac disease or that inflammatory processes can be targeted for cardiac diseases with alterations in iron handling. Studies have shown promise for this method of targeting in cardiovascular disease. For example, deferiprone (DFP), an iron chelator, demonstrated an anti-inflammatory effect on rats with DCM, exhibited by significant decreases in NF-κB and COX2 transcript and protein levels, and rescued cardiomyocyte morphology (Zou et al., 2017). Another example includes a study in which the effects of Canagliflozin (Cana) on ferroptosis in mice with DCM were examined. In this report, Cana reduced inflammatory cell infiltration into myocardial fibers and rescued the excess iron accumulation and glutathione depletion seen in ferroptosis (Du et al., 2022). As mentioned previously, ferroptosis is iron-induced cell death involving a loss of GPx4 activity and a subsequent increase in lipid reactive oxygen species, resulting in lipid peroxidation (Yang and Stockwell, 2016). Other research tested sub-anesthetic doses of Etomidate on attenuation of ferroptosis in a myocardial ischemia/reperfusion rat model. Etomidate treatment significantly reduced expression of pro-inflammatory cytokines and ferroptosis markers, increased NRF2 expression, and rescued LV diastolic and systolic pressure (Lv et al., 2021). Therefore, targeting iron-related processes can affect inflammation and can be useful in treating cardiovascular disease.

One way to target both inflammation and iron metabolism is through the FPN-hepcidin axis. As discussed above, hepcidin regulates the function of FPN found in cardiomyocytes and the cardiovascular system. Antagonizing systemic hepcidin would increase the bioavailability of iron and prevent, for example, the iron deficiency anemia associated with heart failure. Also, altering cellular hepcidin could have an effect from within the tissue by altering iron efflux from barrier cells into underlying tissue. Recently, a hepcidin antagonist has gone through Phase I trials for the treatment of chronic kidney disease and shows promise for future clinical studies (Renders et al., 2019). Chronic kidney disease is known to involve iron deficiency anemia (Stauffer and Fan, 2014), similar to heart failure, therefore this may be a viable treatment option. In the case of cardiovascular diseases with iron excess, decreasing iron availability may be effective. This is being tested with the use of mini-hepcidins, which are small peptides containing 7-9 amino acids from hepcidin that retain its functional activity (Preza et al., 2011). Importantly, mini-hepcidins were shown effective in a mouse model of β-thalassemia by decreasing serum and heart iron levels (Casu et al., 2020). Although more research needs to be completed, targeting the FPN-hepcidin axis may be an effective treatment route for cardiovascular disease.

In conclusion, mechanisms relating to iron handling and inflammatory processes are clearly related, specifically in the cardiovascular system and cardiac disease. The goal of this review was to highlight what is understood about cells of the heart and cardiac disease relating to inflammation and iron handling, with the hope of bringing attention iron metabolism as a therapeutic target for cardiovascular disease. Much about iron handling in cardiomyocytes is understood, but there is a lack of information about the heart barrier and how this responds to systemic iron, which could greatly alter heart iron metabolism. Future research must be completed to fully understand how systemic iron metabolism alters heart iron metabolism. With this greater understanding, therapeutics targeting iron may be utilized to treat inflammatory processes that contribute to cardiovascular disease progression.

## Glossary

Tf: Transferrin
TfR: Transferrin Receptor
DMT1: Divalent Metal Transporter 1
TBI: Transferrin Bound Iron
NTBI: Non-transferrin Bound Iron
FPN: Ferroportin
Cp: Ceruloplasmin
Hp: Hephaestin
APP: Acute Phase Protein
EC: Endothelial Cells
LV: Left Ventricle
LTCC: L Type Calcium Channel
TTCC: T Type Calcium Channel
MI: Myocardial Infarction
ECM: Extracellular Matrix
LVEF: Left Ventricle Ejection Fraction
HF-rEF: Heart Failure with Reduced Ejection Fraction
NOS: Nitric Oxide Synthase
AHA: American Heart Association
ACM: Arrythmogenic Cardiomyopathy
DCM: Diabetic Cardiomyopathy
T2D: Type 2 Diabetes
CRP: C-reactive Protein
RAAS: Renin-Angiotensin-Aldosterone System
TLRs: Toll-Like Receptors
DAMPs: Danger Associated Molecular Patterns
PRRs: Pattern Recognition Receptors
ROS: Reactive Oxygen Species
FHC: Ferritin Heavy Chain
GPX4: Glutathione Peroxidase 4
FRDA: Friedrich’s Ataxia
iPSCs: Induced Pluripotent Stem Cells
ESRD: End Stage Renal Disease
HF-pEF: Heart Failure with Preserved Ejection Fraction
QSM: Quantitative Susceptibility Mapping
Fer-1: Ferrostatin-1
LAD: Left Anterior Descending
IFN-Ɣ: Interferon Gamma
WGCNA: Weighted Gene Co-expression Network Analysis
Lcn2: Lipocalin 2
AGEs: Advanced Glycation End Products
HOX-1: Heme Oxygenase 1
DIC: Doxorubucin-Induced Cardiomyopathy
DFP: Deferiprone
Cana: Canagliflozin
